# The Identification of Patterns in the Relation Between Biodiversity and Mutualistic Ecosystem Function Based on Network Resilience

**DOI:** 10.3390/e27030231

**Published:** 2025-02-24

**Authors:** Changchun Lv, Ye Zhang, Yulin Lei, Ziwei Yuan, Dongli Duan

**Affiliations:** 1 School of Information and Control Engineering, Xi’an University of Architecture and Technology, Xi’an 710311, China; lvcc@xauat.edu.cn (C.L.); yeyez321@outlook.com (Y.Z.); xzz21502@163.com (Y.L.); 2 School of Mechanical Engineering, Northwestern Polytechnical University, Xi’an 710072, China; yuanziwei@mail.nwpu.edu.cn

**Keywords:** complex network, biodiversity, mutualistic ecosystem function, network resilience, redundancy hypothesis

## Abstract

Identifying the relation between biodiversity and mutualistic ecosystem function has been a longstanding concern. In this study, we present an interpretive model to evaluate the impact of each species on mutualistic ecosystem functions. By analyzing network resilience, we derive the average abundance and tipping point of the ecosystem to represent ecosystem functions. Based on the order of species collapse, each species is classified according to the *F*-core. The model quantitatively evaluates the influence of species on mutualistic ecosystem functions in scenarios where species are removed from ecosystems. We propose a criterion for identifying redundant species: a species is considered redundant if its removal negatively impacts average abundance without affecting the tipping point. To validate the model, we introduce twenty-four mutualistic ecosystems. Our numerical simulations and analytical analyses reveal two distinct patterns: one indicating the presence of redundancy and the other suggesting that each species is essential. Additionally, in mutualistic ecosystems characterized by redundancy, specialist species are more likely to be identified as redundant.

## 1. Introduction

Ecosystems are generally characterized as complex systems, and the relation between biodiversity and ecosystem function has been a subject of intense debate among ecologists [1,2]. Biodiversity is often regarded as a key feature that underlies the functioning of ecosystems [3,4,5,6]. However, some experimental and modeling studies have reported weak, negative, or even nonexistent correlations between biodiversity and ecosystem function [1,7]. It is a challenge to decipher the mechanisms that link biodiversity and ecosystem function [8,9].

Cardinale et al. pointed out that the relation between species and ecosystem function can be divided into three patterns: the rivet-redundancy hypothesis, the proportional loss hypothesis, and the immediate catastrophe hypothesis [10]. Hector et al. focused on grassland biodiversity experiments and revealed that different groups of species influence various ecosystem functions, indicating that most species contribute uniquely to ecosystem multifunctionality rather than being redundant [11]. Maestre et al. found a significant positive relation between species richness and multifunctionality. This underscores the non-redundant role of plant biodiversity in enhancing ecosystem functions such as carbon storage, productivity, and nutrient cycling, rather than all species being redundant in these ecosystems [12]. Jing et al. demonstrated that climate mediates the relation between above- and below-ground biodiversity and ecosystem multifunctionality on the Tibetan Plateau, and revealed that both above- and below-ground biodiversity contribute to ecosystem multifunctionality [13]. MacArthur introduced a measure of community stability based on fluctuations in animal populations, and found that the stability is related to the evenness of species abundances within a community [14]. Sasaki et al. revealed a two-phase functional redundancy in Mongolian rangeland plant communities, with high redundancy at low species richness and a plateau at high richness, indicating the non-redundant role of species in maintaining ecosystem functions [15]. Pasari et al. demonstrated that multiple scales of biodiversity, including β diversity, significantly influence ecosystem multifunctionality, with β diversity playing a crucial role in stabilizing multifunctionality across various functions [16]. Grman et al. found that ecosystem multifunctionality in restored prairies is enhanced by β diversity, highlighting the significant role of non-redundant species in maintaining ecosystem functions [17]. Yan et al. investigated the functional diversity of plants and demonstrated that plant functional β-diversity serves as a critical mediator in the impact of drought on soil multifunctionality. This findings highlight the pivotal role of redundant species within plant diversity networks in sustaining soil multifunctionality [18]. Pennekamp et al. showed that biodiversity can increase overall ecosystem resilience when biodiversity is low, and decrease it when biodiversity is high through experiments [19]. Zhang et al. investigated the impact of water depth on microbial communities and ecosystem multifunctionality (EMF) in Lake Hulun, a semiarid lake in China, and found out how climate-induced water depth changes can alter ecosystem structure and functioning [20].

However, the aforementioned study on biodiversity emphasizes experiments aimed at understanding its relation with ecosystem function [21,22]. Previous approaches have not provided precise analytical tools to identify the influence pattern within ecological systems. In other words, there is a lack of an interpretative model to capture the influence of a species on ecosystem function. Complex networks serve as effective tools for capturing the topological properties of ecosystems, while coupled ordinary differential equations can model the interactions between species such as cooperation, competition, and exploitation. Allesina et al. proposed a new criterion for assessing the stability of complex ecological systems based on their network structure [23]. Kundu et al. investigated the sustainability of multilayer ecological networks composed of harvesting patches, revealing the effects of various network structures and connectivity patterns on ecosystem stability and species persistence [24]. Vollert S. A. et al. proposed a novel sequential Monte Carlo sampling method (SMC-EEM) designed to efficiently manage large and complex ecological systems, and this method could facilitate rapid predictions regarding the impacts of conservation measures on endangered species [25]. Recently, Morone et al. proposed a relation between the collapse sequence of species and the k-core structure of networks in mutualistic ecosystems as the strength of mutualistic interactions weakens [26]. Huang et al. conducted an analysis of the identification and stability of critical ecological land in Xinfeng County, a hilly region in southern China. The study assessed various ecological functions, including water conservation, soil and water conservation, biodiversity conservation, and recreational functions, alongside land use status. Utilizing a complex network method, the researchers found that forest land with high vegetation density predominated in critical ecological areas [27]. Si et al. proposed the concept of *F*-core to accurately monitor the process of species or cell extinction as the strength of mutualistic interactions weakens [28]. Although the *F*-core can elucidate the collapse mechanisms of complex systems, it is limited in its ability to identify the relation between biodiversity and ecosystem function.

To address the question, we propose an interpretation model to evaluate the influence of individual species on mutualistic ecosystem functions. Resilience is defined as the ability to adjust activities in order to maintain basic functionality when perturbation occurs [29]. The system may experience a bifurcation or phase transition that leads to a loss of resilience by abruptly shifting to an undesirable fixed point in the equation, commonly referred to as the tipping point [30]. The proposed model identifies the relation pattern between biodiversity and mutualistic ecosystem functions through network resilience, determining whether a species is redundant or a keystone. Here, we introduce the dynamics of an ecosystem with *N* species and test the effectiveness of the interpretation model in real mutualistic networks. The results indicate that the interpretation model is highly effective in predicting relational patterns. We present a general predictive framework to identify ecosystems with redundant species, applicable across a broad range of regions from Europe to Africa. The main contributions of our work are as follows:

1. An interpretive model is introduced to elucidate the influence of biodiversity on mutualistic ecosystem functions. In this model, the average abundance and tipping point, based on network resilience, are derived to represent ecosystem functionality. Species are classified according to the *F*-core.
2. The criterion is introduced to determine whether a species is redundant. Specifically, a species is considered redundant if its removal results in a negative impact on the average abundance, while not influencing the tipping point of the ecosystem.
3. Redundant species are distributed in the low *F*-core, indicating that specialists may be considered redundant species in mutualistic ecosystems.

## 2. The Interpretation Model

The key to the interpretation model is to develop an analytical tool that can assess the influence of each species on the system. The model comprises three questions that need to be addressed: (1) the performance indicators of the system should be identified; (2) each species should be classified; (3) the relation pattern between biodiversity and ecosystem function should be elucidated.

### 2.1. The System Performance Indicator

Ecological modeling is essential for understanding the structure and dynamics of ecosystems, as it provides mathematical frameworks to clarify species interactions, energy flow, and population dynamics. Mutualistic interactions are widely present in ecosystems, such as pollination ecosystems and seed–dispersal ecosystems [31]. The emergence of complex network theory has further advanced the study of ecosystem stability by emphasizing the significance of network topology in determining system robustness. Building upon these foundational concepts, this research will focus on the dynamics of an ecosystem comprising *N* species, with each species *i* defined by its density [28].

(1) $${\dot{x}}_{i}\left(t\right)=-dx_{i}-sx_{i}^{2}+\sum_{j=1}^{N}A_{ij}\gamma_{ij}\frac{x_{i}x_{j}}{\alpha +\sum_{k=1}^{n}A_{ik}x_{k}};$$

this provides a general deterministic representation of an ecosystem driven by mutualistic interactions. In this model, *d* denotes the death rate of a species, and s>0 represents the self-limitation parameter, which accounts for intraspecific competition that restricts the growth of a species once $x_{i}$ surpasses a certain threshold. The symbol α represents the half-saturation constant, $A_{ij}$ is the system’s adjacency matrix, and $\gamma_{ij}$ indicates the strength of the mutualistic interaction between species *i* and *j*.

When the system reaches a steady state, its dynamics can be expressed as

(2) $$-dx_{i}^{*}-sx_{i}^{*2}+\sum_{j=1}^{N}A_{ij}\gamma_{ij}\frac{x_{i}^{*}x_{j}^{*}}{\alpha +\sum_{k=1}^{n}A_{ik}x_{k}^{*}}=0;$$

according to the dimensionality reduction process proposed in the literature [32], when the network reaches the steady state, Equation (2) can be simplified into a one-dimensional dynamic form as follows:

(3) $$-dx_{eff}-sx_{eff}^{2}+\beta_{eff}\gamma_{ij}\frac{x_{eff}^{2}}{\alpha +\beta_{eff}x_{eff}}=0,$$

where $x_{eff}=\langle k\cdot x\rangle /\langle k\rangle \;$ denotes the nearest neighbor weighted abundance, $\beta_{eff}=\langle k^{2}\rangle /\langle k\rangle \;$ denotes the nearest neighbor weighted average degree, $\langle k\rangle$ refers to the average degree of the network, and $\langle k^{2}\rangle$ signifies the second moment of the network’s degree distribution. To simplify the model, we set the parameters α=s=1. The $\gamma_{ij}$ represents the mutualistic interaction strength; it could be simplified to γ for each interaction pair through the adjacency matrix, so the Equation (3) can be simplified to

(4) $$\beta_{eff}x_{eff}^{2}+\left[d\beta_{eff}-\gamma \beta_{eff}+1\right]x_{eff}^{2}+d=0,$$

solving the equation for $x_{eff}$, we can obtain the positive solution as follows

(5) $$x_{eff}=\frac{-d\beta_{eff}+\gamma \beta_{eff}-1+\sqrt{\Delta }}{2\beta_{eff}},$$

where $\Delta =d^{2}\beta_{eff}^{2}+\gamma^{2}\beta_{eff}^{2}+1-2d\beta_{eff}^{2}\gamma^{2}-2d\beta_{eff}-2\gamma \beta_{eff}$. To obtain the abundance of each species, we approximate $x_{j}^{*}$ and $x_{k}^{*}$ as $x_{eff}$ in Equation (2), which simplifies to

(6) $$-dx_{i}^{*}-sx_{i}^{*2}+k_{i}\gamma \frac{x_{i}^{*}x_{eff}}{\alpha +k_{i}x_{eff}}=0,$$

the abundance is only related to its own degree and the weighted abundance of its nearest neighbors; therefore, the steady-state abundance can be expressed as

(7) $$x_{i}^{*}=\frac{k_{i}\left(\gamma_{ij}-d\right)x_{eff}-d}{(1+k_{i}x_{eff})s}$$

where $x_{eff}$ can be obtained through Equation (5).

For evaluating the influence of species on the average abundance of the ecosystem, we consider the removal of a specific fraction (1−f) of nodes. As a result, the structure of the system changes, leading to a modification in the degree distribution of the system

(8) P′(k)=∑k=k′∞P(k)kk′(1−ϖ)k′ϖk−k′.

Further, based on dynamic systems theory, the average abundance of the system is defined as

(9) $$\langle x^{*}\rangle =\sum_{i=1}^{N^{\prime }}\frac{x_{i}^{*}}{N}=\sum_{i=1}^{N^{\prime }}\frac{1}{N}\frac{k_{i}^{\prime }\left(\gamma_{ij}-d\right)x_{eff}^{\prime }-d}{(1+k_{i}^{\prime }x_{eff}^{\prime })s}.$$

The tipping point refers to the phenomenon whereby, when the system’s removal fraction reaches a critical threshold, the nodes within the system become isolated, losing all connections to one another. This indicates that the ecosystem will collapse once it reaches this point. The solution to Equation (4) exists if and only if Δ>0; conversely, when Δ<0, the equation has no solution, and we can conclude that the system has collapsed. Therefore, the critical condition occurs when

(10) $$\Delta =d^{2}\beta_{eff}^{2}+\gamma^{2}\beta_{eff}^{2}+1-2d\beta_{eff}^{2}\gamma^{2}-2d\beta_{eff}-2\gamma \beta_{eff}=0;$$

the tipping point can therefore be determined as

(11) $$\beta_{eff}^{c}=\frac{d+\gamma +\sqrt{d^{2}-\alpha^{2}+4d\gamma }}{\alpha^{2}+\gamma^{2}-2d\gamma }.$$

### 2.2. The Classification of the Species (F-Core)

The ecosystem is a typical complex system that contains a large number of species and their interactions. Identifying the relation between each species and ecosystem functions presents a significant challenge. An effective approach to address this challenge is to classify each species prior to examining the relation between biodiversity and mutualistic ecosystem functions.

The concept of the *F*-core was introduced in complex systems to identify the contribution of each species to the overall system and it can reveal the collapse mechanism [28]. In a mutualistic ecosystem of interacting species, the *F*-core represents the portion of the network that remains after iteratively removing species whose contributions are less than *F*. For a given value of *F*, the subset of species in the *F*-core includes the periphery, called the *F*-shell, and the remaining higher *F*-core. Consequently, the network has a nested structure of *F*-cores with increasing *F*-shells of order $k_{F}$, beginning from the network’s periphery. The low *F*-core encompasses the high *F*-core, and so on, up to the innermost core of the network, which is the maximum *F*-core labeled by the $k_{F-core}^{\max}$. The *F*-core number serves as a topological invariant for each mutualistic ecosystem. In this context, we adopt the concept of the *F*-core, which refers to the largest subgraph that may not be globally connected and includes nodes with strength greater than a threshold *T*.

(12) $$\Psi \left(w-t\right)=\left\{\begin{array}{cc} 0, & \mathrm{if}\;w\leq T,\\ \frac{w-T}{\Delta T}, & \mathrm{if}\;T<w<T+\Delta T,\\ 1, & \mathrm{if}\;w>T+\Delta T. \end{array}\right.$$

The nodes that fall within $F_{shell}=T$ indicate that their contribution to the system shifts from a finite value to zero. In an ecosystem, each species is associated with a strength value *w*, where $w_{i}$ is calculated as $w_{i}=\sum_{j=1}^{N}A_{ij}\Psi \left(w_{j}-T\right)$. The procedure for obtaining the *F*-core structure is as follows:

1. For a specified threshold *T*, we eliminate species with w≤T and retain those with w>T.
2. The contribution of species with T<w≤T+ΔT is (w−T)/ΔT, while other species contribute 1, as described in Equation (12).
3. After completing steps 1 and 2, the values of the species are updated to a smaller $w^{\prime }$. If $w^{\prime }\leq T$ or $T<w^{\prime }\leq T+\Delta T$, steps 1 or 2 are repeated until the contribution of each remaining species stabilizes. This iterative process resembles the algorithm used for extracting the *F*-core from the network.

### 2.3. The Relation Pattern Between Biodiversity and Ecosystem Function

To reveal the relation between biodiversity and ecosystem function, it is essential to determine whether the ecosystem demonstrates redundancy. Two criteria are introduced to identify redundant species within an ecosystem, based on the potential impact of node removal on system performance. The primary focus is on changes in the ecosystem’s average abundance and tipping points. A species is classified as redundant if its removal does not significantly affect the overall performance of the system. The specific criteria for identifying redundant nodes are as follows.

Change in average abundance: The impact of species removal on the average abundance of an ecosystem is a critical consideration. If the removal of a particular species results in an increase in the ecosystem’s average abundance, that species is deemed to have a negative effect on system functionality and may therefore be classified as redundant. In other words, a redundant species must satisfy the following equation:

(13) $$\frac{\langle x^{{*}^{\prime }}\rangle }{\langle x^{*}\rangle }>1,$$

where $\langle x^{{*}^{\prime }}\rangle$ refers to the average abundance of the ecosystem following removal, and $\langle x^{*}\rangle$ refers to its original average abundance.

Change in tipping point: The definition of a redundant species must also consider its impact on the ecosystem’s tipping point. The tipping point refers to the maximum load that the system can sustain or the minimum stability threshold. A redundant species is one that, when removed, does not result in a significant decrease in the system’s tipping point or cause a degradation in system performance. Mathematically, a redundant species must satisfy the following equation:

(14) $$\frac{{\beta_{eff}^{c}}^{\prime }}{\beta_{eff}^{c}}=1,\;$$

where ${\beta_{eff}^{c}}^{\prime }$ denotes the tipping point after removal, and $\beta_{eff}^{c}$ represents the original tipping point.

Thus, two criteria must be met to determine whether a species is redundant. Specifically, a redundant species negatively impacts the average abundance of the ecosystem and does not influence the tipping point. Based on these considerations, the relation pattern between biodiversity and ecosystem function can be elucidated.

## 3. Results

To verify the proposed model, ecological networks were obtained from the open-source website https://www.web-of-life.es/, accessed on 1 January 2025. The parameters for each network are summarized in Table 1. In the subsequent analysis, we set the parameter d=0.5, and the value of γ ranged from 2.5 to 0.6 during the experiment.

From the interpretation model, the average abundance and tipping point are derived as indicators of ecosystem function. Since *F*-core is an effective tool for classifying species, we removed certain species from the network based on their *F*-core values to evaluate their influence on ecosystem function. To ascertain the relation pattern, the following process is employed:

1. To classify species within the network by *F*-core;
2. Removing species based on the sequence of $k_{f}$, the average abundance and the tipping point of the ecosystem are calculated using Equations (13) and (14);
3. The relation patterns are identified for each ecosystem, highlighting both redundant species and keystone species.

Twenty-four mutualistic ecosystems from various continents are presented in Table 1. The following sections provide a detailed examination of two distinct patterns. One pattern highlights the presence of redundancy, whereas the other suggests that each species is essential.

**Figure 1:**
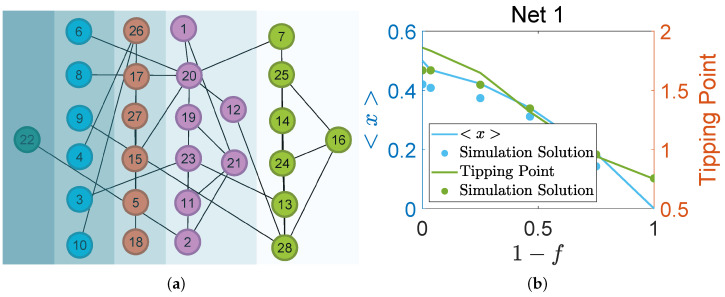
(**a**) *F*-core structure of Network 1. (**b**) The average abundance and tipping point of Network 1 as a function of the node removal fraction.

The schematic diagram of Network 1, classified according to the *F*-core and illustrated in Figure 1a, results in five distinct shells. The names of each species depicted in Figure 1a is listed in Table 2. Node removal based on the *F*-shell numbers leads to variations in the network’s average abundance and tipping point concerning the removal fraction, as shown in Figure 1b. The blue line represents the average abundance, the green line indicates the tipping point, and the dots in the corresponding colors represent the simulation results. The findings demonstrate that when species from each *F*-shell are removed from the network, both the average abundance and the tipping point decline. This indicates that each species contributes positively to the average abundance of the ecosystem, and the absence of any species would result in a decrease in the tipping point. Consequently, Network 1 is considered a non-redundant network.

**Figure 2:**
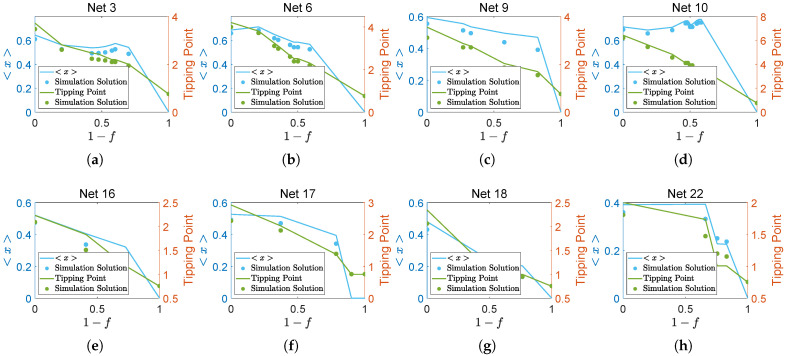
The variation in the average abundance and tipping points of other non-redundant networks.

According to the relation pattern illustrated in Figure 2, we found that eight networks are non-redundant. The blue line represents the average abundance, the green line indicates the tipping point, and the dots in the corresponding colors represent the respective simulation solutions. The results show that when species from each *F*-layer are removed from the network, the tipping point decreases for all eight networks. This indicates that the resilience of the network deteriorates when species are removed from the ecosystem. Regarding average abundance, the ecosystems depicted in Figure 2a–c,e–h exhibit a decrease, while the ecosystem in Figure 2d initially increases before declining. These findings suggest that each species contributes positively to the ecosystem’s functionality.

**Figure 3:**
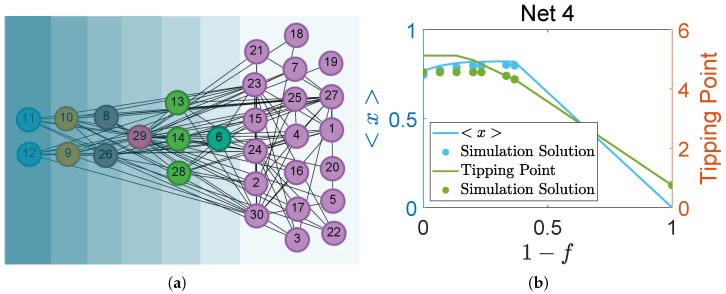
(**a**) *F*-core structure of Network 4. (**b**) The average node abundance and the tipping point of Network 3 as a function of the node removal fraction.

The schematic diagram of Network 4, classified according to the *F*-core, is illustrated in Figure 3a and reveals five distinct shells. The names of each species depicted in Figure 3a are listed in Table 3. Node removal based on the *F*-shell numbers results in variations in the network’s average abundance and tipping point concerning the removal fraction, as shown in Figure 3b. The blue line represents the average abundance, the green line indicates the tipping point, and the dots in the corresponding colors represent the respective simulation solutions. When the node below $k_{f}=2$ is removed, the average abundance shows an increasing trend, while the tipping point remains constant. When nodes with connectivity values between $k_{f}=3.05$ and $k_{f}=3.4$ are removed, the average abundance continues to increase, while the tipping point shows a decreasing trend. However, when the node above $k_{f}=3.4$ is removed, both the average abundance and the tipping point decrease. In other words, as the fraction of removed nodes increases, the average abundance initially rises but later declines, while the tipping point remains constant at first and then decreases. This indicates that the absence of certain species does not significantly impact the system’s functionality. Consequently, Network 4 is considered a redundant network, with redundant species found at $k_{f}=1.01$ and $k_{f}=2$.

**Figure 4:**
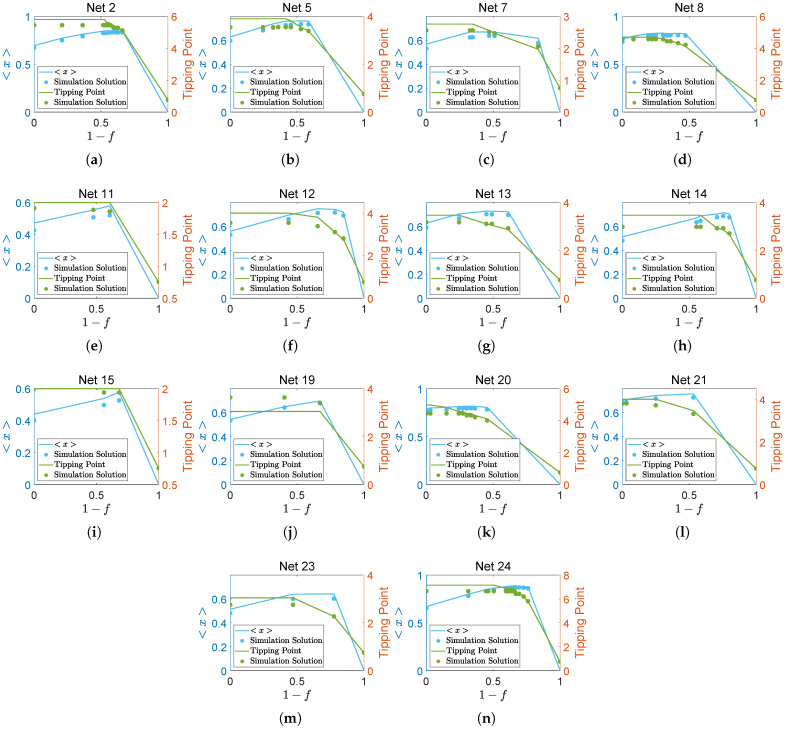
The variation in the average abundance and tipping point of other redundant networks.

According to the relation pattern illustrated in Figure 4, there are redundant species present in 14 networks. The blue line represents the average abundance, while the green line indicates the tipping point. The dots, corresponding in color, represent the respective simulation solutions. The results indicate that when species from each *F*-layer are removed from the network, the average abundance initially rises before declining later, whereas the tipping point remains constant at first and then declines. This suggests that the absence of certain species does not significantly impact the system function. Although some error which exists between analytical solutions and simulation solutions are shown in Figure 4, the phenomenon of redundancy is identified. Specifically, the redundant species in Figure 4a are observed at $k_{f}=1.01$, $k_{f}=2$, $k_{f}=3.05$, and $k_{f}=3.47$. In Figure 4b, the redundant species occur at $k_{f}=1.01$, $k_{f}=1.3$, $k_{f}=1.97$, and $k_{f}=2$. In Figure 4c, the redundant species appear at $k_{f}=1.01$ and $k_{f}=1.78$. In Figure 4d, the redundant species are present at $k_{f}=1.01$, $k_{f}=2$, $k_{f}=2.81$, and $k_{f}=3.05$. In Figure 4e, the redundant species are observed at $k_{f}=1.01$ and $k_{f}=1.36$. In Figure 4f,g, the redundant species occur at $k_{f}=1.01$. In Figure 4h, the redundant species are found at $k_{f}=1.01$ and $k_{f}=1.05$. In Figure 4i, the redundant species appear at $k_{f}=1.01$ and $k_{f}=1.36$. In Figure 4j, the redundant species occur at $k_{f}=1.01$ and $k_{f}=2$. In Figure 4k, the redundant species are present at $k_{f}=2$. In Figure 4l, the redundant species appear at $k_{f}=1.01$ and $k_{f}=2$. In Figure 4m, the redundant species are observed at $k_{f}=1.01$. Finally, in Figure 4n, the redundant species occur at $k_{f}=1.01$, $k_{f}=2$, $k_{f}=2.2$, and $k_{f}=3.05$.

**Figure 5:**
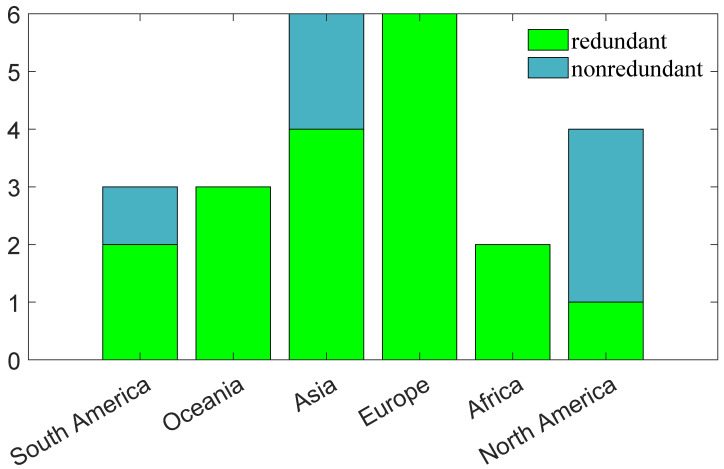
The distribution of redundant networks across various continents.

To further analyze the spatial distribution characteristics of redundant networks, Figure 5 illustrates the distribution of these networks across various regions. This distribution map offers a clear visual representation of the number of redundant networks in each area, aiding in the identification of regional differences in redundancy occurrence. The results indicate that ecosystems in Oceania, Europe, and Africa contain redundant species, while certain ecosystems in other continents are non-redundant.

**Figure 6:**
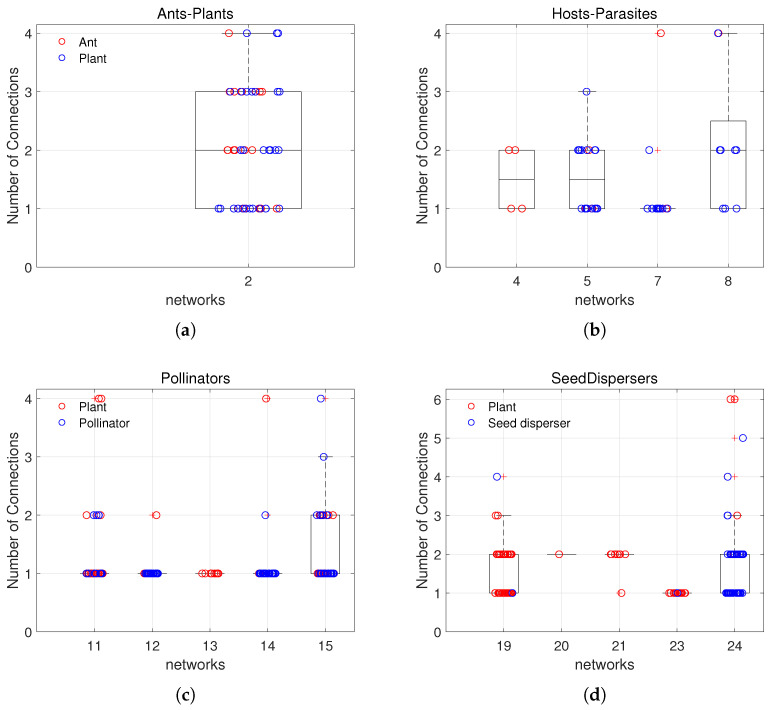
(**a**) The distribution of connection numbers among redundant species in ant–plant networks is illustrated. Red circles represent ant species, while blue circles represent plant species. (**b**) The distribution of connection strengths among redundant species in host–parasite networks is shown, with red circles indicating host species and blue circles indicating parasite species. (**c**) The distribution of connection strengths among redundant species in pollination networks is depicted, where red circles represent plant species and blue circles represent pollinator species. (**d**) The distribution of connection strengths among redundant species in seed–dispersal networks is presented, with red circles denoting plant species and blue circles representing seed-disperser species.

From the results presented in Figure 6, all redundant species are located in the low *F*-core. The characteristics of these redundant species warrant further analysis. In an ecosystem, a specialist refers to a species that relies on a limited number or specific types of resources—such as particular pollinators or host plants—and often exhibits highly specialized structures or behaviors to adapt to these stable conditions. In contrast, a generalist interacts with a wide variety of species, utilizing a broad range of resources and adopting flexible, opportunistic strategies to thrive in diverse environments [33,34,35,36]. In this context, we consider species with a higher number of connections (greater than or equal to 3) as highly interactive and classify them as generalists, while species with fewer connections (less than 3) are classified as specialists. The nature of the connection between plants and animals can be assessed to determine whether the species are specialists or generalists. Figure 6 illustrates the distribution of redundant species across various mutualistic ecosystems.

Figure 6a illustrates the distribution of connection number among redundant species in the ant–plant network. Red circles represent ant species, while blue circles represent plant species. Overall, the distribution of connections among redundant species is broad, ranging from a minimum of 1 to a maximum of 4, indicating the presence of both specialists, which form connections with a limited number of species, and generalists, which maintain connections with a broader range of species. The majority of species have a connection number of 1 or 2, suggesting the specialist characteristic, while a small proportion exhibit higher connection number of 3 or 4, indicating the generalist characteristic.

Figure 6b illustrates the distribution of the connection numbers among redundant species within four host–parasite networks. Red circles represent host species, while blue circles denote parasitic species. In Network 4, all species have a connection number of 1 or 2, indicating that they all species exhibit the specialist characteristic. In Networks 5, 7, and 8, the majority of species also have a connection number of 1 or 2, suggesting a similar specialist characteristic. However, in Network 5, one parasitic species has a connection number of 3; in Network 7, one host species has a connection number of 4; and in Network 8, one parasitic species has a connection number of 4. These particular species exemplify the generalist characteristic.

Figure 6c illustrates the distribution of connection number among redundant species across five pollination networks. Red circles represent plant species, while blue circles denote pollinator species. In these networks, the connection number among redundant species is predominantly concentrated in the low range (connection number = 1 or 2), indicating that these redundant species primarily function as specialists, forming connections with only a limited number of species. In Networks 11 and 14, a small number of plant species exhibit slightly higher connection strengths (connection number = 4), suggesting the presence of the specialist characteristic. Network 15 displays a higher median value and a broader interquartile range, with several highly connected species (connection number = 3 or 4), further indicating the generalist characteristic among these species.

Figure 6d illustrates the distribution of connection number among redundant species in five seed–dispersal networks. Red circles represent plant species, while blue circles denote seed–disperser species. In Networks 19 and 24, the connection number among redundant species exceeds 3. Notably, in Network 24, several species exhibit a connection number reaching 5 or 6, indicating the presence of generalist species in both networks. These generalists establish links with multiple other species and may play a crucial role in maintaining the structural stability of the network. In contrast, Network 20 features only one redundant plant species with a connection number of 2, classifying it as a specialist. In Network 21, the connection numbers of redundant species are concentrated at 1 and 2, all of which are specialists. In Network 23, all redundant species have a connection number of 2, displaying the specialist characteristic.

## 4. Discussion

We propose an interpretative model to reveal the relation between biodiversity and the functioning of mutualistic ecosystems. The average abundance and the tipping point, based on network resilience, are derived to represent ecosystem functionality. Species are classified according to the order of extinction using the *F*-core metric. To assess whether a species is redundant, it is sequentially removed from the ecosystem, starting from the lowest to the highest *F*-core value. We analyze the microscopic changes in average abundance for each species, as well as the macroscopic changes in the ecosystem’s tipping point. A species is considered redundant if its removal negatively impacts average abundance while having no effect on the tipping point.

A total of 24 mutualistic ecosystems are introduced to verify the feasibility of the proposed model. Two distinct patterns are found in different mutualistic ecosystems. The first pattern indicates that each species is indispensable and that the extinction of each species would result in a decrease in both the average abundance and the tipping point of the ecosystem. The second pattern highlights the existence of redundancy, wherein certain species negatively impact the average abundance of the ecosystem. Ecosystem types also affected the relation pattern in mutualistic ecosystems. Pollination networks and seed–dispersal networks are more likely to have redundant species than host–parasite networks. The observed results suggest that in real-world mutualistic networks the redundancy phenomenon also exists [10], consistent with the result in this paper. It is observed that redundant species are predominantly located within the low *F*-core, and their characteristics are subsequently analyzed. The results show that the connection number of most redundant species is below 3. Moreover, the connection number is only 1 for 67.6% of redundant species. Consequently, specialists are identified as the redundant species within mutualistic ecosystems. As mentioned previously [36,37], this may be due to the fact that specialist species are sensitive to environmental changes, while generalist species are more adaptable. Morone et al. [26] pointed out that the species would be more likely to be extinct in a low k-core when the interaction between species decreased. Therefore, it is important to note that redundant species are not entirely devoid of ecological value; rather, they may serve as indicators of impending ecosystem collapse.

Based on the above discussion, the present analysis has several limitations. For example, although the relation pattern between biodiversity and mutualistic ecosystem function was identified, there are some errors between the theoretical result and the simulation result for the approximation used to derive the function of the ecosystem. An important condition for the applicability of the present analysis is that the ecosystem must be mutualistic. However, this work did not examine systems which consider the condition of positive and negative interactions. These interactions are common in real ecosystems, in the form of, for example, cooperation and competition in ecological networks [38,39,40]. Moreover, while the result only applies to local ecosystems and the structure of the ecosystem is unchanged, a restricted understanding of temporal interaction dynamics is a more serious limitation. To avoid this limitation, connecting these patterns to human activities or climate change should be carried out [31]. In the future, these limitations should be considered.

## Figures and Tables

**Table 1:** Network parameters of ecosystems.

Network	Type	Dimension	Continent	Average Degree	Weight
1	Ants–Plants	39	South America	2.3	Weighted
2	Ants–Plants	91	Oceania	6.5	Weighted
3	Hosts–Parasites	42	Asia	4.6	Weighted
4	Hosts–Parasites	32	Asia	6.7	Weighted
5	Hosts–Parasites	53	Asia	4.6	Weighted
6	Hosts–Parasites	36	Asia	5.4	Weighted
7	Hosts–Parasites	49	Asia	3.6	Weighted
8	Hosts–Parasites	36	Europe	7.1	Weighted
9	Hosts–Parasites	58	Asia	3.7	Weighted
10	Hosts–Parasites	62	Europe	7.3	Weighted
11	Plants–Pollinators	61	South America	2.7	Unweighted
12	Plants–Pollinators	78	Europe	3.7	Weighted
13	Plants–Pollinators	49	Europe	4.3	Unweighted
14	Plants–Pollinators	65	Africa	3.2	Weighted
15	Plants–Pollinators	66	South America	2.5	Unweighted
16	Plants–Pollinators	29	North America	2.6	Weighted
17	Plants–Pollinators	72	North America	3.2	Weighted
18	Seed–Dispersers	38	North America	2.6	Weighted
19	Seed–Dispersers	79	Oceania	3.6	Unweighted
20	Seed–Dispersers	33	Europe	7.3	Unweighted
21	Seed–Dispersers	32	North America	5.4	Unweighted
22	Seed–Dispersers	61	Oceania	2.2	Unweighted
23	Seed–Dispersers	49	Europe	3.1	Weighted
24	Seed–Dispersers	121	Africa	6.9	Weighted

**Table 2:** *F*-core of each species in Network 1.

*F*-Core	$k_{f}=0.98$	$k_{f}=1.01$	$k_{f}=1.69$	$k_{f}=1.83$	$k_{f}=1.86$
	㉒ Duroia saccifera	③ Allomerus prancei	⑤ Azteca sp1	① Allomerus auripunctata	⑦ Azteca sp2
		④ Azteca polymorpha	⑮ Crematogaster sp4	② Allomerus octoarticulatus	⑬ Crematogaster sp3
		⑥ Azteca sp1	⑰ Pheidole minuta	⑪ Crematogaster sp1	⑭ Crematogaster sp4
**Species**		⑧ Azteca sp3	⑱ Pseudomyrmex concolor	⑫ Crematogaster sp2	⑯ Pheidole minutula
		⑨ Azteca sp4	㉖ Tachigali myrmecophila	⑲ Pseudomyrmex nigrescens	㉔ Maieta guianensis
		⑩ Azteca sp5	㉗ Tachigali polyphylla	⑳ Cecropia purpurascens	㉕ Maieta poeppiggi
				㉑ Cordia nodosa	㉘ Tococa bullifera
				㉓ Hirtella myrmecophila	

**Table 3:** *F*-core of each species in Network 4.

*F*-Core	Species
$k_{f}=1.01$	⑪ Microtus oeconomus ⑫ Microtus oeconomus
$k_{f}=2$	⑨ Microtus agrestis ⑩ Microtus arvalis
$k_{f}=3.05$	⑧ Micromys minutus ㉖ Hystrichopsylla talpae
$k_{f}=3.47$	㉙ Neopsylla mana
$k_{f}=4$	⑬ Neomys fodiens ⑭ Neomys fodiens ㉘ Megabothris turbidus
$k_{f}=4.33$	⑥ Clethrionomys rutilus
$k_{f}=5.12$	① Apodemus agrarius ② Apodemus speciosus ③ Arvicola terrestris ④ Clethrionomys glareolus
	⑤ Clethrionomys rufocanus ⑦ Cricetus cricetus ⑮ Sorex Araneus ⑯ Sorex arcticus
	⑰ Sorex caecutiens ⑱ Sorex daphaenodon ⑲ Sorex isodon ⑳ Sorex minutissimus
	㉑ Sorex minutus ㉒ Talpa europaea ㉓ Amalaraeus penicilliger ㉔ Ctenophthalmus assimilis
	㉕ Frontopsylla elata ㉙ Megabothris rectangulatus ㉚ Palaeopsylla soricisk

## Data Availability

All data are contained within the article.
